# Tailored Phytochitosomes as Targeted Nanotherapy for Alveolar Bone Regeneration in Diabetic Obese Rats

**DOI:** 10.3390/ph19030506

**Published:** 2026-03-19

**Authors:** Yosra S. R. Elnaggar, Mariam Zewail, Eman M. Salem, Wafaa Y. Alghonemy, Nevien M. Ahmed, Rania A. Hanafy, Waiel Daghistan, Ali M. Alaseem, Dina Khodeer, Elsayed G. Zaki, Ahmad N. Almougy, Mona A. Moustafa

**Affiliations:** 1 Department of Pharmaceutics, College of Pharmacy, Imam Abdulrahman Bin Faisal University, Dammam 31441, Saudi Arabia; 2 Department of Pharmaceutics, Damanhur University, Damanhur 22511, Egypt; 3 Department of Oral Biology, Faculty of Dentistry, Pharos University, Alexandria 21565, Egypt; 4 Department of Basic Dental Sciences, Faculty of Dentistry, Zarqa University, Zarqa 13110, Jordan; 5 Department of Oral Biology, Faculty of Dentistry, Tanta University, Tanta 31773, Egypt; 6 Department of Oral Biology-Biochemistry, Faculty of Dentistry, Pharos University, Alexandria 21565, Egypt; 7 Department of Dental Material, Faculty of Dentistry, Pharos University, Alexandria 21565, Egypt; 8 Department of Anesthesia and Surgery, College of Medicine, Imam Mohammad Ibn Saud Islamic University (IMSIU), Riyadh 11432, Saudi Arabia; 9 Department of Pharmacology, College of Medicine, Imam Mohammad Ibn Saud Islamic University (IMSIU), Riyadh 13317, Saudi Arabia; 10 Material Science Department, Egyptian Petroleum Research Institute, Cairo 11727, Egypt; 11 Department of Material Science, Institute of Graduate Studies and Research, Alexandria University, Alexandria 21565, Egypt; 12 Department of Pharmaceutics, Faculty of Pharmacy, Pharos University, Alexandria 21565, Egypt

**Keywords:** luteolin, chitosan, alveolar socket healing, diabetes mellitus, obesity, bone regeneration

## Abstract

**Background/Objectives**: Individuals with diabetes often experience difficulties in the healing of their alveolar sockets. Furthermore, obesity is strongly associated with the development and progression of type 2 diabetes through complex metabolic and inflammatory mechanisms. The current study provides new insights into the use of Luteolin (LU) and/or chitosan vesicles (CHV) to accelerate bone regeneration, highlighting a biologically and clinically relevant approach that leverages implants as a clinical solution. **Methods**: Sixty rats were randomly categorized into five groups: Group I (negative control); Group II (positive control), diabetic and obese rats; Group III (LU-treated), diabetic and obese rats with an extraction socket loaded with LU; Group IV (CHV-treated), diabetic and obese rats with an extraction socket loaded with CHV; and Group V (LU-CHV), diabetic and obese rats with an extraction socket loaded with LU-CHV. After 2 and 6 weeks, rats’ mandibles underwent histological, histomorphometric, biochemical, and statistical analyses. **Results**: The results demonstrated significant differences among the experimental groups. The LU-CHV formulation showed superior therapeutic performance compared with free luteolin and the untreated control group. In vitro release studies revealed sustained, controlled release from LU-CHV, whereas free luteolin exhibited rapid drug release. Additionally, LU-CHV significantly enhanced biological activity, as evidenced by improved anti-inflammatory and/or therapeutic markers compared to the other groups. These findings indicate that encapsulation within chitosan vesicles improved drug stability, bioavailability, and overall therapeutic efficiency. **Conclusions**: LU-CHV demonstrated superior efficacy compared to free luteolin, highlighting the advantage of chitosan-based vesicular delivery systems. LU-CHV not only enhanced controlled drug release and therapeutic outcomes but also presents a promising platform that could significantly advance targeted drug delivery strategies in inflammatory and metabolic disorders. The findings suggest that LU-CHV represents a transformative approach in improving treatment effectiveness and patient outcomes.

## 1. Introduction

Tooth extraction socket healing follows a tightly regulated cascade of inflammatory, proliferative, and remodeling phases that culminate in new bone formation [1]. This process can be markedly disrupted in systemic metabolic disorders such as uncontrolled diabetes mellitus and obesity, where persistent inflammation, oxidative stress, and vascular impairment compromise bone regeneration. Consequently, developing locally targeted therapeutic strategies capable of modulating both inflammatory and osteoclastogenic pathways is essential to improve healing outcomes under metabolically compromised conditions [2,3].

DM and obesity are characterized by persistent low-grade systemic inflammation with elevated circulating pro-inflammatory cytokines, including TNF-α, IL-1β, and IL-6. All these cytokines increase osteoclastogenesis by upregulating receptor activator of nuclear factor kappa B ligand (RANKL) and suppressing its decoy receptor, osteoprotegerin (OPG) [4]. RANKL, expressed by osteoblasts and stromal cells, binds to its receptor RANK on osteoclast precursors, promoting their differentiation, activation, and survival, whereas OPG competitively inhibits this interaction. In diabetic and obese states, the increased RANKL/OPG ratio enhances excessive osteoclast activity and bone resorption, disrupting normal bone remodeling [5]. Furthermore, hyperglycemia, accumulation of advanced glycation end products (AGEs), oxidative stress, and impaired angiogenesis negatively affect osteoblast differentiation and matrix mineralization [6]. Collectively, inflammation-driven RANKL/OPG imbalance combined with osteoblast dysfunction compromises bone regeneration and delays healing in metabolically impaired conditions.

Chitosan (CH), a naturally derived polysaccharide, has gained considerable attention in regenerative applications due to its biodegradability, biocompatibility, mucoadhesive properties, and intrinsic wound-healing potential [7,8]. Importantly, CH can be employed as a functional coating material for vesicular nanocarriers, enhancing colloidal stability, prolonging local retention, and enabling sustained drug release. Liposomal systems, in particular, offer efficient drug encapsulation and controlled delivery; however, their clinical performance in extraction sockets is limited by rapid dispersion and insufficient site-specific retention. Surface modification with chitosan overcomes these limitations by improving physicochemical stability and maintaining prolonged therapeutic concentrations at the target site [9,10,11,12]. Luteolin (LU), a bioactive flavone, exhibits potent anti-inflammatory, antioxidant, and anti-osteoclastogenic activities, particularly under diabetic conditions, through regulation of oxidative and inflammatory signaling pathways [13,14]. Nevertheless, its therapeutic efficacy is hindered by poor aqueous solubility and limited bioavailability.

To the best of our knowledge, this is the first study to employ luteolin-loaded chitosan-coated vesicles as a localized, non-antibiotic nanotherapeutic to enhance post-extraction socket healing and bone regeneration under combined diabetes–obesity conditions, thereby directly addressing a clinically challenging scenario for subsequent implant rehabilitation.

## 2. Results

### 2.1. Confirmation of the Diabetic–Obese Rat Model

The successful establishment of the diabetic–obese model was confirmed by significant increases in body weight and fasting blood glucose levels in the experimental animals compared with the control group. At the end of the induction period, the diabetic–obese rats exhibited a significantly higher mean body weight (298 ± 11 g) compared with the control group (185 ± 8 g, *p* < 0.05) (Table 1).

Similarly, fasting blood glucose levels were markedly elevated in the diabetic–obese group (312 ± 28 mg/dL) compared with controls (92 ± 6 mg/dL; *p* < 0.001), confirming the successful induction of hyperglycemia. These findings validate the establishment of the diabetic–obese animal model used in the present study (Table 2).

### 2.2. Characterization of LU-Loaded Chitosan Vesicles (LU-CHV)

#### 2.2.1. PS, ZP, and %EE

As Table 3 demonstrates, efficient CH coating in the formulation LU-CHV as the particle size increased from 88.92 ± 1.14 to 220.71 ± 2.70 upon coating (Student’s *t*-test *p* < 0.05). In addition, the zeta potential value was shifted from −20.05 ± 1.56 to 23.60 ± 2.78 after CH coating. PDI of LU-blank and LU-CHV were 0.22 ± 0.05 and 0.31 ± 0.13, respectively, suggesting their monodispersity and homogeneity [15].

Based on the overhead results, the prepared LU-CHV exhibited suitable zeta potential and percent EE values (23.60 ± 2.78 mV and 92.33 ± 1.14%, respectively) comparable to those of the hydrophobic drug LU.

LU-CHV demonstrated colloidal stability upon storage for 6 months at 4 °C with no significant changes in PS, ZP, and EE% % (Student’s *t*-test *p* < 0.05).

#### 2.2.2. Morphological Examination Using TEM

**Figure 1:**
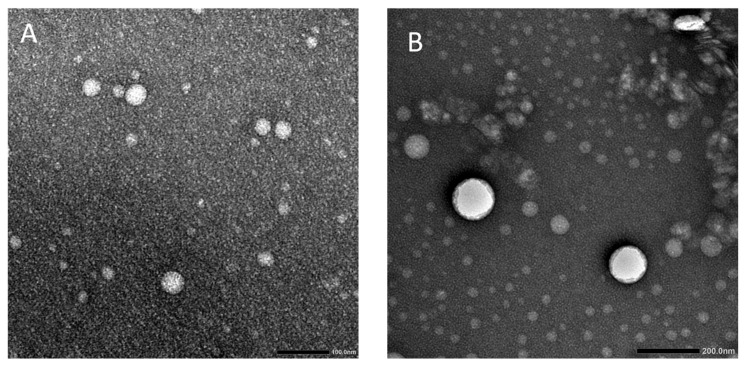
TEM micrographs of: (**A**) LU-blank magnified at 100 nm and (**B**) LU-CHV magnified at 200 nm.

As illustrated in Figure 1, the prepared LU-CHV had a spherical uniform shape with no signs of aggregation or coalescence. The CH coating layer is discrete; it appears as a dark coating adjacent to the particles and is more evident than in the blank, uncoated formulation. Although TEM micrographs are shown at different magnifications, all scale bars are clearly indicated, and these magnifications were chosen to optimally display both overall morphology and fine structural details of the samples.

#### 2.2.3. In Vitro Release Behavior

**Figure 2:**
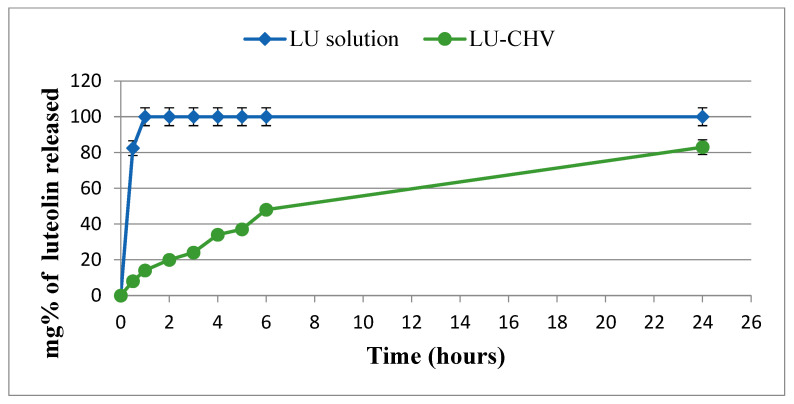
LU release from LU-encapsulated CHV using dialysis bag system in PBS (7.4) at 37 °C. Results are attained as the mean ± SD.

The in vitro release profile of luteolin (LU) from LU-loaded chitosan vesicles (LU-CHVs) was evaluated in phosphate-buffered saline (PBS, pH 7.4) containing 0.5% Tween 80 and compared with that of free LU solution. Free LU demonstrated rapid drug liberation, with cumulative release reaching approximately 80% and 100% within 30 and 60 min, respectively, reflecting the absence of a diffusion barrier. In contrast, LU-CHVs exhibited a limited initial burst release of 8% and 14% at 30 and 60 min, respectively. This early release phase is attributed to the diffusion of surface-associated drug molecules. In contrast, the subsequent phase was characterized by sustained, controlled release over 24 h, consistent with a biphasic release pattern (Figure 2). The sustained release behavior underscores the capacity of the chitosan-coated vesicular system to modulate drug diffusion and prolong therapeutic availability.

### 2.3. Biocompatibility Test

**Figure 3:**
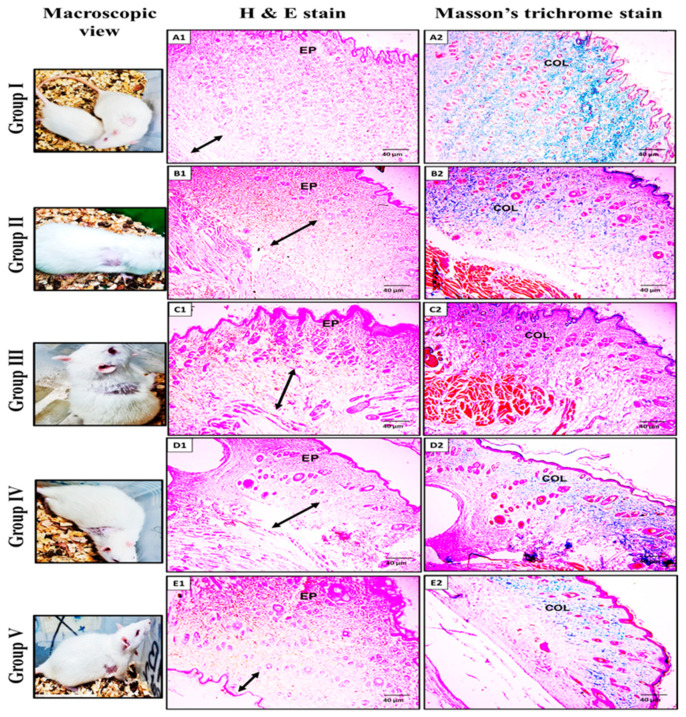
A macroscopic view of rats and LM micrographs of the full thickness of the skin of all experimental groups. In the macroscopic view, rat skin appears intact in all groups, with no visible signs of erythema, edema, ulceration, or necrosis. (**A1**,**B1**,**C1**,**D1**,**E1**) H&E-stained sections, (**A2**,**B2**,**C2**,**D2**,**E2**) Masson’s trichrome-stained sections. (**A1**,**A2**) Group I (negative control) showing thickened epithelial (EP) layer, normal thickness of subcutaneous fatty layer (doubled arrow), and normal thickness and distribution of collagen (COL) fibers (blue staining). (**B1**,**B2**) group II (positive control), (**C1**,**C2**) group III (LU-treated), (**D1**,**D2**) group IV (CHV-treated), and (**E1**,**E2**) group V (LU-CHV), all sections showing thickened subcutaneous fatty layer (doubled arrow), with the thinner one depicted at group V, all of them showing nearly the same EP thickness. Also, COL appears to have a normal distribution and thickness. (**A1**,**B1**,**C1**,**D1**,**E1**) (H& E stain ×100). (**A2**,**B2**,**C2**,**D2**,**E2**) (Masson’s trichrome stain ×100).

The macroscopic view of the rats’ skin appeared intact in all groups, with no visible signs of erythema, edema, ulceration, or necrosis. Histologically, Group I (negative control) showed a thickened epithelial layer, normal thickness of the subcutaneous fatty layer, and normal thickness and distribution of collagen fibers. Group II (positive control), group III (LU-treated), group IV (CHV-treated), and group V (LU-CHV) showed a thickened subcutaneous fatty layer, with the thinnest one depicted at group V. All of them showed nearly the same epithelial thickness. Also, collagen appeared with normal distribution and thickness (Figure 3).

The statistical analysis confirmed that the EP thickness and collagen distribution in Group V did not differ significantly (*p* > 0.05) from those in the negative control (Group I), indicating near-complete histological recovery. For the Subcutaneous Fatty Layer: While Groups II, III, and IV remained significantly thicker than the negative control, Group V exhibited a significant reduction in subcutaneous thickness compared to the positive control (Group II) (*p* < 0.01), marking a distinct advancement in the tissue remodeling phase (Supplementary Table S3).

### 2.4. Light Microscopic Results

#### H&E Stain Results

**Figure 4:**
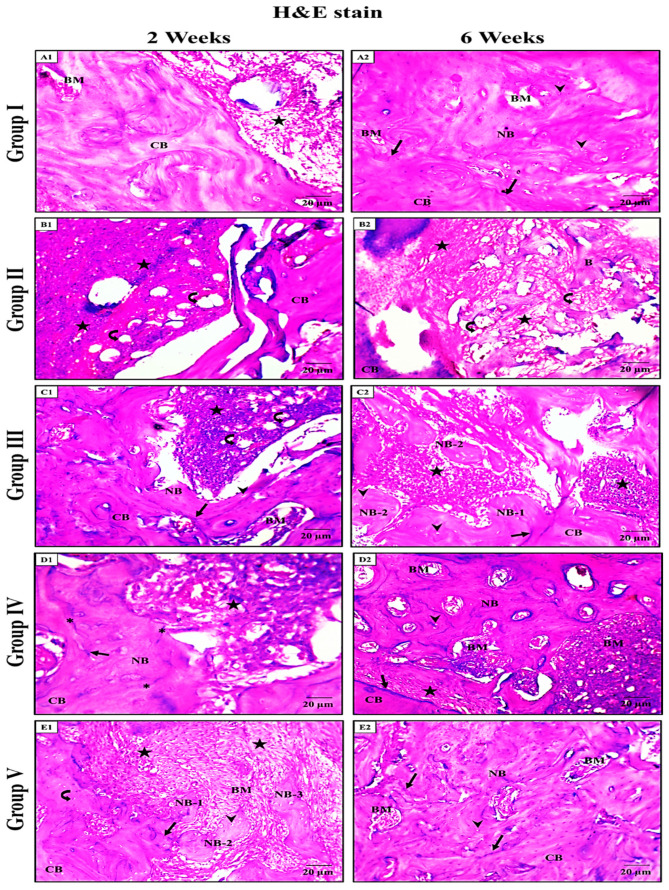
LM micrographs of H&E-stained sections for the extraction socket in all experimental groups. (**A1**,**A2**) group I (negative control) at 2 and 6 weeks, respectively. (**A1**) fibrous granulation tissue (stars) fills the socket space near the cortical bone (CB) of the socket wall. (**A2**) the newly formed bone (NB) fills the extraction socket and contains many large osteocytes (OS) (arrowhead) and vascular bone marrow (BM) spaces, which differ from the mature CB of the socket wall. The densely stained line (arrow) lines the NB from the native CB of the socket wall. (**B1**,**B2**) group II (positive control) for 2 and 6 weeks, respectively. (**B1**) Dense granulation tissue (stars) with fat cells (curved arrows) plugs the socket area that the CB of the socket wall surrounds. (**B2**) dense granulation tissue (stars) filled with fat cells (curved arrows) plugs the socket area, with thin bone trabeculae (B) that the CB of the socket wall surrounds. (**C1**,**C2**) group III (LU-treated) at 2 and 6 weeks, respectively. (**C1**) dense granulation tissue (stars) with fat cells (curved arrow) plugs into the socket area. Dense Bone resting line between the NB, which contains prominent, large-sized OS (arrow heads), and the CB of the socket wall, which includes smaller-sized OS. (**C2**) the socket is filled with dense fibrous granulation tissue (stars) and thin bone trabeculae of NB-2, which contain many prominent, large-sized OS (arrowheads). The remodeling line (arrow) lines the NB-1 from the CB of the socket wall. (**D1**,**D2**) group IV (CHV-treated) at 2 and 6 weeks, respectively. (**D1**) Granulation tissue (star) with the NB fills part of the extraction socket, which differs from the mature CB of the socket wall. A dense bone resting lines (*), which indicate active bone formation areas. The densely stained line (arrow) lines the NB from the native CB of the socket wall. (**D2**) the socket is filled with thin bone trabeculae of NB with a small area of dense fibrous granulation tissue (stars). The NB contains many prominent, large-sized OS (arrowhead) and large BM spaces. The remodeling line (arrow) lines the NB from the CB. (**E1**,**E2**) group V (LU-CHV) at 2 and 6 weeks, respectively. (**E1**) Dense granulation tissue (stars) plugs into the socket area. Dense Bone resting line between the newly formed bone (NB-1), which contains prominent, large-sized OS (arrow heads), and the CB of the socket wall, which includes smaller-sized OS (curved arrow). Notice the single bony masses formed inside the granulation tissue (NB-2 and NB-3). (**E2**) the NB filling the extraction socket contains many large OS (arrowhead), and vascular BM spaces, which differ from the mature CB of the socket wall. The densely stained line (arrow) indicates areas of active bone formation (H& E stain ×400).

Group I (negative control):

At two weeks, fibrous granulation tissue was detected, filling the socket space near the cortical bone (CB) of the socket wall (Figure 4(A1)).

At six weeks, a newly formed bone was identified, filling the extraction socket and containing many large osteocytes (OS) with vascular bone marrow (BM) spaces, which differ from the mature CB of the socket wall. The densely stained lines appeared to line the newly formed bone from the native CB of the socket wall (Figure 4(A2)).

Group II (positive control):

At two weeks, as in the negative control, some dense granulation tissue, with fat cells, was observed, plugging the socket area that the CB of the socket wall surrounded (Figure 4(B1)).

At six weeks, as in the previous interval, dense granulation tissue with fat cells still appeared to be plugging the socket area, but with thin bone trabeculae that the CB of the socket wall surrounded (Figure 4(B2)).

Group III (LU-treated):

At two weeks, as in the same interval for group II, a dense granulation tissue with fat cells appeared, filling the socket area. Also, a dense resting line was present between the newly formed region, which contained prominent, large OS, and the CB of the socket wall, which contained smaller OS (Figure 4(C1)).

At six weeks, the socket was filled with both dense fibrous granulation tissue and thin trabeculae of newly formed bone, with many prominent, large OS. In addition, the remodeling lines were depicted between the newly formed bone and the CB of the native socket wall (Figure 4(C2)).

Group IV (CHV-treated):

At two weeks, both the granulation tissue and the newly formed bone-filled part of the extraction socket were present. The new bone differed histologically from the mature CB of the socket wall. Additionally, dense bone resting lines appeared, indicating areas of active bone formation (Figure 4(D1)).

At six weeks, the socket was filled with thin bone trabeculae of newly formed bone, with a larger amount than in the previous interval of the same group, and a small area of dense fibrous granulation tissue. The newly formed bone contained many prominent, large OS and BM spaces, with remodeling lines distributed throughout the bone (Figure 4(D2)).

Group V (LU-CHV):

At two weeks, the socket area appeared to be filled with dense granulation tissue adjacent to the single bony mass. Dense bone resting lines were noticed between the newly formed bone, which contained prominent, large-sized OS, and the native bone of the socket wall, which contained smaller-sized OS (mature bone) (Figure 4(E1)).

At six weeks, not as the same interval as all other groups, the newly formed bone was noticed filling the extraction socket, which contained many large OS, and vascular BM spaces, which differ from the mature CB of the socket wall, with a densely stained line indicating active bone formation areas (Figure 4(E2)).

Collectively, in the Initial Phase (Week 2), the healing process was predominantly characterized by fibrous granulation tissue filling the socket space adjacent to the native cortical bone. In Group I and Group V, early signs of osteogenesis were evident, with newly formed bone containing large, prominent osteocytes. Notably, Group V exhibited unique isolated bony masses within the granulation tissue, suggesting an accelerated osteogenic induction compared to Group II, which remained filled with dense granulation tissue and fat cells (Figure 4).

In the Maturation Phase (Week 6), the granulation tissue was replaced mainly by newly formed bone and vascular bone marrow spaces in the most successful groups. A distinct remodeling line was observed in Groups I, III, IV, and V, demarcating the boundary between the mature native and the regenerated bone. While Group II still showed a prevalence of fat cells and only thin bone trabeculae, Group V demonstrated nearly complete socket filling with mature, vascularized bone, mirroring the architecture of the negative control (Figure 4) (Table 4).

### 2.5. Statistical Results of Biochemical Analysis

#### 2.5.1. Inflammatory Cytokines (IL-6, TNF-α and IL-1β)

At 2 weeks, Group II (positive control) exhibited a pronounced inflammatory response compared with Group I (negative control) as evidenced by marked elevations in IL-6 (83.63 ± 10.90 vs. 32.50 ± 5.93 pg/mg protein), TNF-α (89.88 ± 10.09 vs. 31.38 ± 6.09 vs. pg/mg protein), and IL-1β (61.88 ± 10.08 vs. 26.50 ± 6.12 pg/mg protein). Groups III, IV, and V demonstrated marked elevations in inflammatory cytokines as follows: IL-6 (65.50 ± 10.53 vs. 68.25 ± 7.36 vs. 43.13 ± 7.22 pg/mg protein, respectively), TNF-α (63.38 ± 8.88 vs. 65.38 ± 8.90 vs. 47.38 ± 7.19 pg/mg protein, respectively), and IL-1β (46.63 ± 7.44 vs. 48.38 ± 8.47 vs. 32.50 ± 5.68 pg/mg protein, respectively).

**Figure 5:**
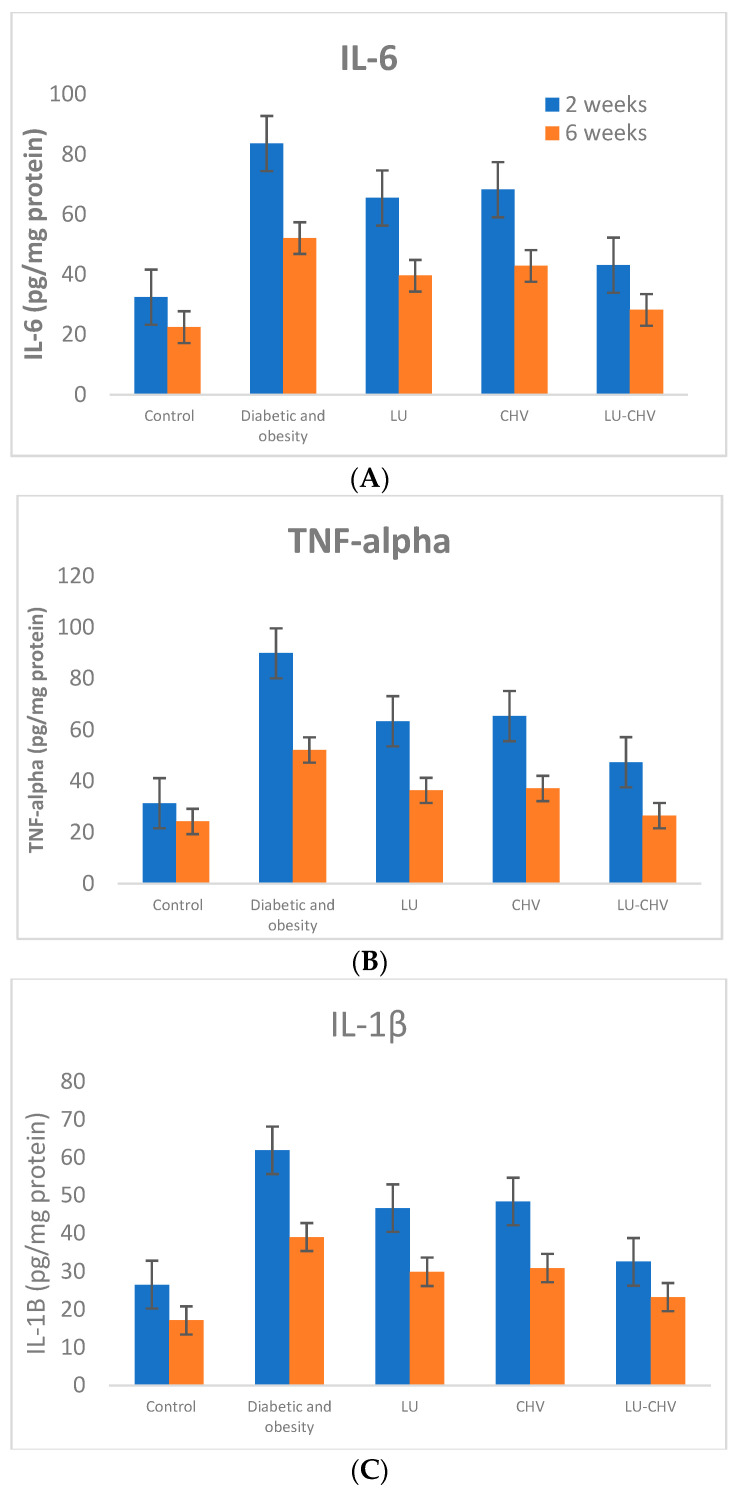
(**A**) Effects of luteolin (LU), chitosan (CHV), and their combination (LU–CHV) on tissue inflammatory IL-6 in diabetic-obese rats at 2 and 6 weeks. Values are expressed as mean ± SD. (**B**) Effects of luteolin (LU), chitosan (CHV), and their combination (LU–CHV) on tissue inflammatory TNF-alpha in diabetic-obese rats at 2 and 6 weeks. Values are expressed as mean ± SD. (**C**) Effects of luteolin (LU), chitosan (CHV), and their combination (LU–CHV) on tissue inflammatory IL-1β in diabetic-obese rats at 2 and 6 weeks. Values are expressed as mean ± SD. Data are presented as mean ± SD (*n* = 8). Statistical analysis was performed using one-way ANOVA followed by Tukey’s post hoc test for multiple comparisons. *p* ≤ 0.05 was considered statistically significant.

At six weeks, these increases persisted, with IL-6, TNF-α, and IL-1β remaining significantly higher in the positive control than in the negative control values (52.13 ± 6.96 vs. 22.50 ± 3.66, 52.13 ± 7.75 vs. 24.25 ± 4.62, and 39.0 ± 8.11 vs. 17.13 ± 3.0 pg/mg protein, respectively). Groups III, IV, and V demonstrated a time-dependent reduction in inflammatory markers, with Group V (LU-CHV) showing the most substantial attenuation at both time points with reduced IL-6, TNF-α, and IL-1β levels to 20 ± 4, 26 ± 5, and 14 ± 3 pg/mg protein, respectively, approaching values observed in the healthy control of Group I (Figure 5A–C) (Supplementary Table S4).

#### 2.5.2. Osteogenic Marker (Osteocalcin)

**Figure 6:**
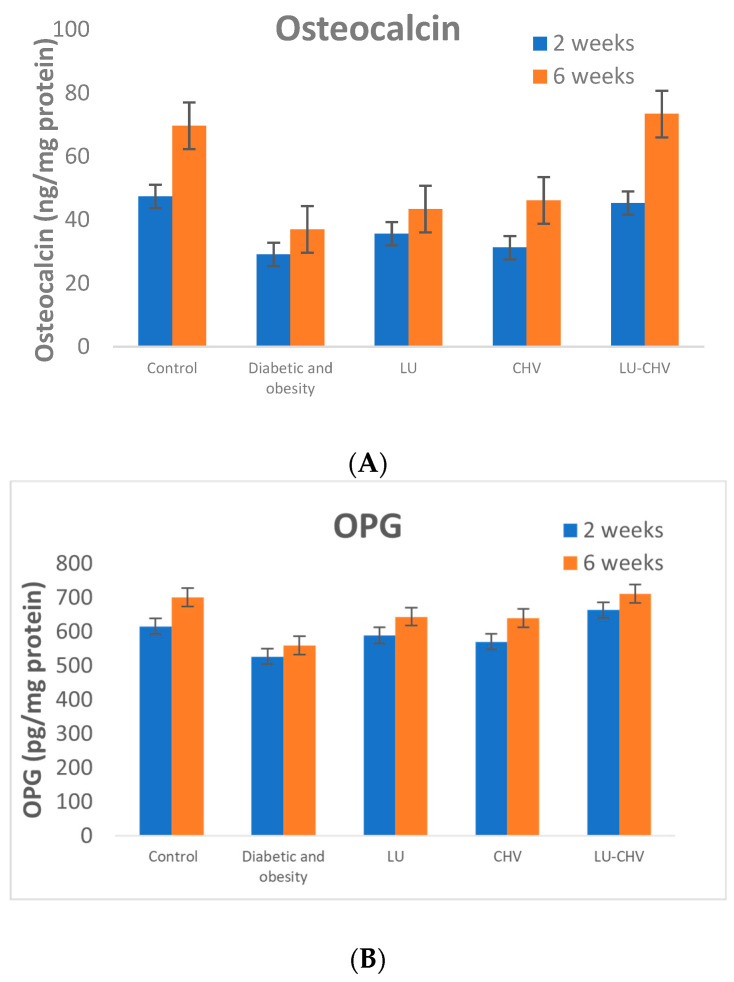

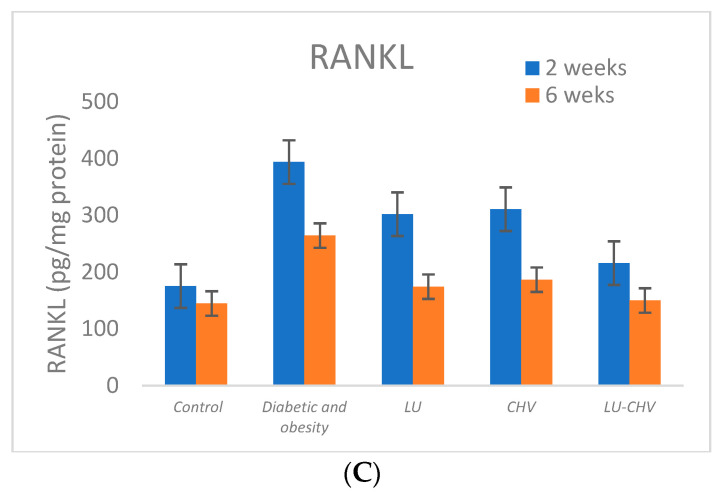
(**A**) Effects of luteolin (LU), chitosan (CHV), and their combination (LU–CHV) on tissue osteocalcin in diabetic-obese rats at 2 and 6 weeks. Values are expressed as mean ± SD. (**B**) Effects of luteolin (LU), chitosan (CHV), and their combination (LU–CHV) on tissue OPG in diabetic-obese rats at 2 and 6 weeks. Values are expressed as mean ± SD. (**C**) Effects of luteolin (LU), chitosan (CHV), and their combination (LU–CHV) on tissue RANKL in diabetic-obese rats at 2 and 6 weeks. Values are expressed as mean ± SD. Data are presented as mean ± SD (*n* = 8). Statistical analysis was performed using one-way ANOVA followed by Tukey’s post hoc test for multiple comparisons. *p* ≤ 0.05 was considered statistically significant.

Osteocalcin levels were significantly lower in Group II (positive control) at both two and six weeks, indicating reduced osteogenic signaling. At two weeks, Osteocalcin decreased from 47.38 ± 8.60 pg/mg protein in Group I (negative control) to 29.13 ± 5.59 pg/mg protein in Group II (positive control). Both Group III (LU-treated) and Group IV (CHV-treated) showed partial increases in Osteocalcin expression (35.63 ± 6.37 and 31.25 ± 5.95 pg/mg protein, respectively). In contrast, Group V (LU-CHV) exhibited a more pronounced increase in osteocalcin levels, reaching 45.25 ± 9.16 pg/mg protein, which closely approximated the negative control value. At six weeks, Osteocalcin decreased from 69.63 ± 10.06 pg/mg protein in Group I (negative control) to 37.0 ± 5.55 pg/mg protein in Group II (positive control). Both Group III (LU-treated) and Group IV (CHV-treated) showed partial increases in Osteocalcin expression (43.38 ± 9.84 and 46.13 ± 10.51 pg/mg protein, respectively). In contrast, Group V (LU-CHV) exhibited a more pronounced increase in osteocalcin levels, reaching 73.38 ± 8.70 pg/mg protein, which closely approximated the negative control value. This progressive decline across treatment groups reflects a graded suppression of osteoblast-promoting activity (Figure 6A) (Supplementary Table S5).

#### 2.5.3. Osteoprotegerin (OPG)

At two weeks, OPG levels were moderately reduced in Group II (positive control) compared with Group I (negative control) (527.1 ± 58.63 vs. 616.0 ± 59.17 pg/mg protein). Both Group III (LU-treated) and Group IV (CHV-treated) restored OPG levels toward normal values (589.4 ± 51.30 and 570.6 ± 83.60 pg/mg protein, respectively). Notably, Group V (LU-CHV) yielded the highest OPG concentration (663.6 ± 68.18 pg/mg protein),

At six weeks, OPG levels were moderately reduced in Group II (positive control) compared with Group I (negative control) (560 ± 90 vs. 720 ± 100 pg/mg protein). Both Group III (LU-treated) and Group IV (CHV-treated) restored OPG levels toward normal values (644.4 ± 45.63 and 639.9 ± 48.33 pg/mg protein, respectively). Notably, Group V (LU-CHV) yielded the highest OPG concentration (712.3 ± 45.94 pg/mg protein), surpassing both Group II and Group I. This elevation suggests a favorable shift toward inhibition of osteoclastogenesis in the combined LU-CHV treatment group (Figure 6B) (Supplementary Table S5).

#### 2.5.4. Osteoclastogenic Marker (RANKL)

RANKL levels were significantly elevated in Group II (positive control) at both two and six weeks, indicating enhanced osteoclastogenic signaling.

At 2 weeks, RANKL increased from 175.3 ± 19.65 pg/mg protein in Group I (negative control) to 393.8 ± 31.02 pg/mg protein in Group II (positive control). Both Group III (LU-treated) and Group IV (CHV-treated) partially reduced RANKL expression (301.9 ± 38.17 and 310.8 ± 32.23 pg/mg protein, respectively), whereas Group V (LU-CHV) produced a more pronounced reduction, lowering RANKL levels to 215.8 ± 36.12 pg/mg protein.

At 6 weeks, RANKL increased from 144.6 ± 7.25 pg/mg protein in Group I (negative control) to 264.3 ± 18.25 pg/mg protein in Group II (positive control). Both Group III (LU-treated) and Group IV (CHV-treated) partially reduced RANKL expression (174.3 ± 12.83 and 186.6 ± 13.91 pg/mg protein, respectively), whereas Group V (LU-CHV) produced a more pronounced reduction, lowering RANKL levels to 149.9 ± 16.27 pg/mg protein, closely approximating the negative control value. This progressive decline across treatment groups reflects a graded suppression of osteoclast-promoting activity (Figure 6C) (Supplementary Table S5).

#### 2.5.5. RANKL/OPG Ratio

The RANKL//OPG ratio (Table 5A,B), a sensitive indicator of bone remodeling balance, was markedly increased in Group II (positive control) at six weeks compared with Group I (negative control), reflecting a dominant osteoclastogenic environment. Both Group III (LU-treated) and Group IV (CHV-treated) had reduced the ratio. Notably, Group V (LU-CHV) demonstrated complete normalization of the RANKL//OPG ratio, matching the healthy control of Group I.

### 2.6. Statistical Results of Bone Histomorphometry

**Figure 7:**
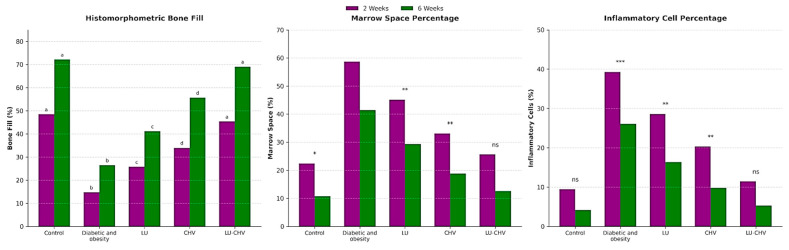
Bar chart showing the histomorphometric analysis of bone surface area %, Marrow Space Percentage, and Inflammatory Cell Count (%). Data are presented as mean ± SD. Statistical analysis was performed using one-way ANOVA followed by Tukey’s post hoc test for multiple comparisons. *p* ≤ 0.05 was considered statistically significant. * *p* ≤ 0.05, ** *p* ≤ 0.001 and *** *p* ≤ 0.0001. Different letters indicate statistically significant differences (*p* ≤ 0.05).

For bone surface area, a significant effect of LU-CHV was detected in group V at both 2 and 6 weeks. Additionally, no significant differences were detected between Group I (negative control) and Group V (LU–CHV) at a two-week interval (*p* = 0.0002). Still, the difference did not persist after six weeks (*p* = 0.2069), while Group II (positive control) was significantly lower than all others (*p* < 0.0001) (Supplementary Table S6) (Figure 7).

For bone-marrow spaces percentages and inflammatory cells, they were also highly significant (*p* < 0.0001), and Group II (positive control) was significantly worse than all others (*p* < 0.0001). Group I and Group V were not significantly different in either the two- or six-week time points, as follows: bone marrow space percentages at two weeks, *p* = 0.8000; at six weeks, *p* = 0.6789. Inflammatory cells percentages at two weeks, *p* = 0.6420; at six weeks, *p* = 0.9973 (Supplementary Tables S6–S8) (Figure 7).

## 3. Discussion

Phospholipids are widely utilized in nanoformulations owing to their amphiphilic structure and excellent biocompatibility, which facilitate efficient drug encapsulation and delivery [16]. Ethanol was selected as the solvent because of its lower toxicity relative to conventional organic solvents and its ability to enhance membrane flexibility, thereby improving vesicle deformability. Tween 80 served as an edge activator, disrupting lipid packing and reducing interfacial tension, which promotes the formation of highly deformable vesicles with smaller particle sizes and improved drug delivery performance.

Bioadhesive nanosystems have recently gained attention for their capacity to enhance localized drug delivery. Chitosan-coated liposomes (CHVs) exploit electrostatic interactions between positively charged chitosan and negatively charged lipid membranes, forming a stable coating reinforced by hydrogen bonding. This modification improves colloidal stability, mucoadhesion, and controlled release properties, thereby extending drug residence time at target sites. These characteristics are reflected in the present findings, in which LU-CHVs demonstrated sustained drug release compared with free LU, supporting the functional advantage of chitosan coating in modulating diffusion kinetics and enhancing localized therapeutic exposure. These results align with the literature, which reports that polymer-coated vesicular systems improve stability and prolong bioavailability through controlled-release mechanisms [17,18].

LU-CHV represents a promising platform for targeted drug delivery in alveolar bone regeneration. Although clinical translation remains limited, the combined attributes of mucoadhesion, controlled release, and biocompatibility provide a strong rationale for its application in oral regenerative therapy. The enhanced therapeutic performance observed in this study can be attributed to the interaction between the positively charged chitosan surface and negatively charged mucin within oral tissues, which promotes electrostatic adhesion and increases nanosystem residence time. This interaction minimizes drug washout from the gingival crevicular fluid, thereby maintaining higher local LU concentrations and improving therapeutic efficacy. Such findings are consistent with previous reports demonstrating that mucoadhesive nanocarriers enhance localized drug retention and bioactivity [17].

Delayed socket healing in metabolically compromised conditions, such as diabetes and obesity, poses a significant clinical challenge, particularly in implant dentistry. Direct delivery of therapeutic agents to the extraction socket enhances bioavailability and optimizes regenerative outcomes. Embedding LU within a chitosan-based liposomal system integrates controlled release with improved tissue retention, addressing limitations of conventional delivery approaches. The observed enhancement in bone formation in the CHV-treated group can be attributed to chitosan’s osteoconductive and wound-modulating properties, which provide a biocompatible platform that supports cellular migration and tissue organization. Its cationic nature facilitates interactions with negatively charged extracellular matrix components, promoting cell adhesion and early regenerative activity. These effects are consistent with prior evidence indicating that chitosan-based formulations enhance tissue repair by stabilizing the wound environment and modulating inflammatory responses [19].

Histological findings further support the role of CHV in improving bone regeneration, as evidenced by increased trabecular bone formation and the presence of prominent osteoblasts at six weeks. This aligns with chitosan’s osteoconductive capacity to guide mineralization and structural organization of newly formed bone. However, the persistence of residual granulation tissue and thinner trabeculae suggests that CHV alone may be insufficient for optimal regeneration in cases of severe metabolic impairment. This observation underscores the potential need for adjunctive osteoinductive strategies to enhance bone healing further and support clinical applications, such as immediate implant placement in compromised sockets [20]. Accordingly, combining chitosan-based delivery systems with bioactive or osteogenic agents may represent a more comprehensive therapeutic approach for improving regenerative outcomes in high-risk patients.

While these systemic antibiotics and NSAIDs post-extraction provided a controlled baseline for healing, the significant differences observed in the subcutaneous layer thickness and collagen area fraction in Group V (LU-CHV) suggest that the regenerative effects of the LU-CHV treatment are independent of the standard pharmacological protocol. Specifically, Group V showed a significantly thinner subcutaneous layer compared to Group II, despite both groups receiving the same systemic medications. This indicates that the LU-CHV scaffold actively modulates the tissue microenvironment beyond the generalized anti-inflammatory effects of NSAIDs.

Many preclinical studies support the notion that LU has potent bone-protective properties, which direct human mesenchymal stem cells to differentiate into osteoblasts. This resulted in accelerated osteogenesis and organized matrix deposition, making it an appealing candidate for enhancing alveolar bone regeneration. Beyond its effects on bone cells, LU also plays a crucial role in the healing environment, facilitating the reparative state. This dual regulation is particularly valuable in conditions of impaired socket healing, where excessive ROS and persistent inflammation disrupt bone formation [21,22].

Moreover, the present study demonstrates that the diabetic–obese condition induces a sustained pro-inflammatory microenvironment within peri-extraction tissues, as reflected by the marked elevations in TNF-α, IL-6, and IL-1β at both experimental time points. The persistence of these cytokines over six weeks indicates a failure of inflammation resolution, a hallmark of impaired bone healing in metabolic disorders. TNF-α and IL-6 are well known to stimulate osteoclast differentiation and suppress osteoblast activity, while IL-1β amplifies local inflammatory signaling and bone resorption. Similar cytokine profiles have been reported in diabetic and obese models, where chronic low-grade inflammation compromises bone regeneration and delays socket healing [23]. In parallel with the inflammatory rise, the significant upregulation of RANKL observed in group II highlights enhanced osteoclastogenic signaling. As RANKL is a central mediator of osteoclast differentiation and activation, its elevation directly contributes to excessive bone resorption. The partial reduction in RANKL levels following individual treatments indicates some therapeutic benefit. However, the combined formulation in group V achieved near-complete normalization. This finding is particularly relevant, as previous studies have emphasized that effective suppression of RANKL is critical for restoring bone remodeling balance in compromised healing environments [24,25]. Conversely, OPG, the endogenous decoy receptor of RANKL, exhibited a slight reduction in Group II, thereby further disrupting the remodeling equilibrium in favor of osteoclastic bone resorption. The combined treatment not only restored OPG expression to baseline levels but also significantly elevated it above control levels, indicating active suppression of osteoclastogenesis. The concurrent upregulation of OPG and marked downregulation of RANKL resulted in substantial normalization of the RANKL/OPG ratio. As this ratio is recognized as a more sensitive and integrative biomarker of bone metabolic status than either parameter alone, its restoration to near-control levels provides robust biochemical evidence of therapeutic efficacy [26].

**Figure 8:**
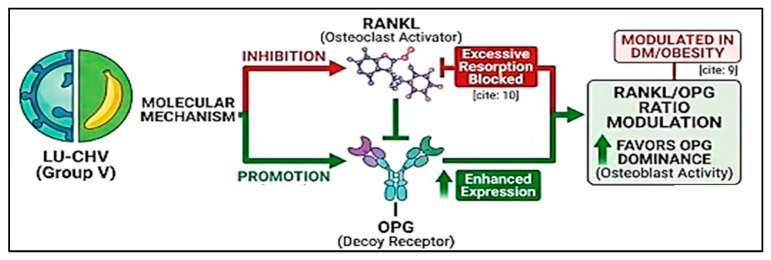
Biological Mechanism of RANKL/OPG for Bone Formation.

Importantly, normalization of the RANKL/OPG ratio paralleled a pronounced reduction in pro-inflammatory cytokines, suggesting a close association between inflammatory modulation and bone remodeling processes. However, as the present findings are derived from biomarker measurements rather than direct pathway analyses, these observations should be interpreted as indicative correlations rather than definitive mechanistic confirmation. Chronic inflammation is known to enhance RANKL expression while suppressing OPG production, thereby linking immune dysregulation to accelerated skeletal deterioration. The present findings suggest that the combined formulation may contribute to improved healing through modulation of inflammatory and osteoclastogenic biomarkers, ultimately re-establishing a microenvironment favorable for bone regeneration (Figure 8). These observations are consistent with recent evidence indicating that effective bone repair in metabolically compromised conditions necessitates simultaneous attenuation of inflammation and inhibition of osteoclast activity, rather than selective targeting of a single pathway [27]. Although the present findings demonstrate significant reductions in inflammatory cytokines and improvements in osteogenic biomarkers following LUT–CHS treatment, these results are based on biomarker measurements. Therefore, the proposed mechanisms should be interpreted as associative rather than definitive, and further molecular studies are needed to elucidate the precise signaling pathways involved.

Notably, metabolically compromised patients, such as those with DM or obesity, exhibit significantly higher rates of implant failure due to delayed bone healing and impaired soft tissue integration [28]. Thus, the local administration of LU-CHV to a diabetic obese person can effectively bridge the healing gap caused by chronic systemic inflammation and impaired microcirculation, as it might serve as a bio-booster that counteracts the hostile physiological environment caused by DM and obesity.

Based on these considerations, we hypothesized that encapsulating LU within chitosan-coated vesicles (LU-CHVs) would generate a synergistic therapeutic effect by integrating sustained local drug delivery with simultaneous suppression of inflammation and osteoclastogenesis. Through coordinated modulation of oxidative stress and bone remodeling pathways, this nanoformulation is expected to restore a regenerative microenvironment conducive to enhanced socket healing and bone formation in metabolically impaired conditions.

It is important to acknowledge that systemic antibiotic and NSAID administration may have influenced the inflammatory outcomes measured in this study. Both are known to possess immunomodulatory and anti-inflammatory effects. While these agents were necessary to manage post-surgical infection risk and pain, their potential to cause stunning effects should be considered when interpreting the inflammatory marker data. Nevertheless, the overall patterns observed across experimental groups remain consistent with the study’s primary objectives, supporting the validity of the conclusions.

Accordingly, the present study offers new insights into the application of natural, non-antibiotic nanocarriers to accelerate bone and soft-tissue regeneration, emphasizing a biologically relevant and clinically translatable strategy that supports implant-based therapy. Notably, the coexistence of diabetes mellitus (DM) and obesity substantially compromises post-extraction socket healing, thereby challenging subsequent implant rehabilitation [11,12].

## 4. Materials and Methods

### 4.1. Ethical Approval

All experimental procedures were conducted in accordance with the guidelines established by the Research Ethics Committee of the Faculty of Pharmacy, Pharos University, Alexandria, Egypt (Ethical Code: PUA0220251223446). Animal handling and care were handled in accordance with the ARRIVE guidelines.

### 4.2. Materials

Lipoid^®^ S100 (l-α-phosphatidylcholine) was purchased from Lipoid AG (Ludwigshafen, Germany). Cholesterol and chitosan (Degree of deacetylation 85%) were obtained from Sigma-Aldrich (Steinem, Germany). The remaining chemicals are analytical grade.

### 4.3. Preparation of LU-Encapsulated Chitosan Vesicles (LU-CHV)

Chitosan (CH) coating was carried out using the reported titration technique [29]. For the preparation of drug-loaded liposomes, luteolin (LU, 7.5 mg/mL) was first dissolved in ethanol together with the lipid phase. Subsequently, the ethanolic phospholipid solution was added dropwise to 10 mL of distilled water using a syringe while stirring at 900 rpm. Then, the mixture was ultrasonicated for 2 min at 60% amplitude using a Sonica R 2200 EP S3 (Soltec, Molina de Segura, Murcia, Spain).

### 4.4. Characterization of LU-CHV

#### 4.4.1. Particle Size (PS), Zeta Potential (ZP) and %EE Measurements

Particle size, polydispersity index (PDI), and zeta potential were determined using a Zetasizer Nano ZS (Malvern Instruments, Worcestershire, UK) [15]. Entrapment efficiency of the prepared LU-CHVs was assessed by cooling centrifugation to separate the free drug. The amount of unencapsulated LU was quantified spectrophotometrically at 350 nm [15]. Blank formulations were analyzed in parallel to eliminate any interference from formulation components in the UV absorbance measurements.

#### 4.4.2. Morphological Analysis

Following 1% uranyl acetate staining, the morphology of the selected formulation was assessed using a transmission electron microscope (TEM) (JEM-1400; JEOL, Tokyo, Japan) [15].

#### 4.4.3. In Vitro Release of LU

The in vitro release of LU from both the solution and optimized formulation F5 was evaluated using dialysis bags (MWCO 12,000–14,000) in phosphate buffer (pH 7.4) with 0.5% Tween 80 at 37 °C and 100 rpm [15]. Samples were withdrawn at set intervals up to 24 h, replaced with fresh medium to maintain sink conditions, and LU content was measured spectrophotometrically at 350 nm. Experiments were performed in triplicate, and results are expressed as mean ± SD.

#### 4.4.4. Stability Study

A six-month stability study was performed by storing the formulation at 4 °C in sealed glass containers. Assessments included visual inspection for sedimentation or phase separation, particle size, zeta potential, and drug leakage (expressed as entrapment efficiency, EE%) [30].

### 4.5. In Vivo Experiments

#### 4.5.1. Sample Size Calculation

The sample size was determined based on a previous study [31]. The minimum sample size was 12 rats per group, resulting in a total of 60 specimens (12 × 5 = 60 animals). This prediction was based on a standardized effect size of 0.814 and a power of 80% (β = 0.20) at an α level of 0.05. To eliminate ambiguity, a computer-generated sequence was used, and a precise random allocation method was employed, with random numbers determining treatment status. The subject and all study team members were oblivious to the treatment distribution. To ensure regular group sizes, any samples lost during the experiment were restored.

#### 4.5.2. Group Assignment and Animal Preparation

Sixty healthy adult male albino rats (Rattus norvegicus, Wistar strain), (6–9 weeks old, 200–250 g) with intact teeth were obtained from the Pharos University animal center. Fourteen days before the study, rats were screened to ensure they were free of systemic or oral diseases. During the experiment, they had ad libitum access to food and water and were housed under controlled lighting and temperature in accordance with ARRIVE guidelines and institutional animal welfare regulations [32]. A total of 60 rats were randomly categorized into five groups (*n* = 12 rats per group):

Group I (negative control): healthy rats without any intervention or treatment

Group II (positive control) diabetic and obesity induced rats, with tooth extraction, and the extraction socket was left without treatment.

In Group III (LU-treated) diabetic and obese rats, following tooth extraction, the extraction socket was loaded with LU.

Group IV (CHV-treated) diabetic and obesity induced rats, with tooth extraction, and the extraction socket was loaded with CHV.

Group V (LU-CHV) diabetic and obese rats underwent tooth extraction, and the extraction socket was loaded with LU-CHV.

#### 4.5.3. Induction of Obesity

A total of 48 rats (from groups II, III, IV, and V) became obese by consuming a highly palatable, high-fat diet (HFD) for 6 weeks. HFD was composed of 40% sheep fats blended with standard food, as reported by Kotańska M. et al. [33] (Supplementary Table S1).

#### 4.5.4. Induction of DM

DM was induced in a total of 48 rats (from groups II, III, IV, and V) by injection of a single intraperitoneal dose of streptozotocin (STZ) (50 mg/kg body weight, freshly reconstituted in 0.1 M citrate buffer, pH of 4.5) (Sigma-Aldrich, St. Louis, MO, USA). Blood glucose levels (BGL) were evaluated three days post-fasting using a digital glucometer (i-SENS, Inc., Seoul, Republic of Korea). Rats were considered diabetic when fasting BGL exceeded 250 mg/dL, in accordance with established criteria [34]. During the experimental period, BGL were consistently monitored to sustain the diabetic condition (Supplementary Table S2).

#### 4.5.5. Tooth Extraction Procedure

Before the tooth extraction, the diabetic obese rats underwent fasting with free access to water for one to two hours. Then the animals received an intramuscular (IM) injection of atropine sulfate at a dosage of 0.4 mL/kg to decrease salivary flow during the procedures. The general anesthesia was induced through injection of an IM mix of 10% ketamine hydrochloride (Ketamine Alfasan 10%, Woerden, The Netherlands) and 2% xylazine (Adwia, 10th of Ramadan City, Egypt), at doses of 0.2 mL/kg and 0.5 mL/kg body weight, respectively [35]. Bilateral extraction of the mandibular first molars was performed according to Moghadam et al. [36]. Firstly, an iodine swab was applied to the area of the lower first molars. Afterwards, each tooth was luxated using surgical elevators by tipping it slowly in the buccal and lingual directions for one second each. This procedure was repeated 10 times until the tooth was luxated, and then it was extracted.

In groups II, III, IV, and V, the sockets were loaded with the proposed materials, and then the sockets were sutured with 4-0 black silk sutures [37]. All rats were then administered Cataflam (IM) (Novartis, Cairo, Egypt) every 8 h for 2 days and Ampicillin 25 mg/kg (Misr Co., Cairo, Egypt) three times daily for 5 days based on body weight. After 2 and 6 weeks of lower first molar extraction, six rats in each group were euthanized, respectively.

#### 4.5.6. In Vivo Biocompatibility Test

Evaluation of biocompatibility and degradation rate is crucial for assessing the safety and efficacy of biomaterials. After general anesthesia, as mentioned before, subcutaneous implants of LU, CHV, and LU-CHV were placed in six rats of Group III, Group IV, and Group V, respectively. However, a clean-cut wound without implant application was performed in groups I and II. The rats were placed in a supine position with the dorsal neck region shaved and disinfected by povidone-iodine solution. Afterwards, a midline incision, approximately 4 cm, was made on the dorsal neck region using a sterile scalpel. Then, materials were implanted, and all the incisions were closed with surgical sutures. Animals were then monitored postoperatively for any signs of distress or adverse reactions against the implanted membranes. Rats were observed for 6 weeks to evaluate the degradation rate of the specimens and to assess any external signs of irritation or inflammation. After rats were euthanized humanely, the skin tissue surrounding the implantation area was dissected and prepared for H&E and Masson’s trichrome histological examination.

#### 4.5.7. Histological Evaluation and Histomorphometric Analysis

After mandibular dissection, the right halves of each mandible were prepared for light microscopic (LM) assessments. In accordance with the specified protocol, tissues were decalcified, fixed in 10% formalin, and embedded in paraffin blocks. After sectioning the materials to 5 μm, staining was performed with hematoxylin and eosin (H&E). Histological investigation was conducted by two histologists using LM with a Leica ICC50 HD digital camera to capture and label figures of distinctive regions.

The captured images were subjected to morphometric analysis using ImageJ version 1.53h17 (National Institutes of Health, Bethesda, MD, USA) to assess the percentage of new bone formation in all groups [38]. For each specimen, three non-overlapping fields were evaluated at ×400 magnification, and the mean percentage of bone area (bone area/total socket area × 100) was calculated. Two blinded examiners performed all measurements, and the resulting mean values were used for statistical analysis.

#### 4.5.8. Biochemical Analysis

Following euthanasia, peri-extraction soft tissues in the left halves of each mandible were carefully excised, rinsed with ice-cold phosphate-buffered saline (PBS) to remove residual blood, and immediately processed. Tissues were accurately weighed and homogenized in PBS at 4 °C to obtain uniform tissue suspensions. Tissue homogenates were centrifuged at 4 °C to remove cellular debris, and supernatants were stored at −80 °C, with analyses conducted after a single freeze–thaw cycle. Biomarker levels were normalized to total protein and expressed as pg/mg protein. Bone remodeling markers—including Osteoprotegerin (OPG), RANKL, osteocalcin, and sclerostin—were quantified using rat-specific ELISA kits (Elabscience, Houston, TX, USA; Catalog No. MBS2022619), while inflammatory status was assessed by measuring IL-6, IL-1β, and TNF-α using commercially available rat ELISA kits (Catalog Nos. E-EL-R0012, E-EL-R0015, and E-EL-R2856, respectively). Quantitative data were expressed as range (minimum and maximum), mean, and standard deviation One way ANOVA test was used for comparing the different studied groups, followed by Post Hoc test (Tukey) for pairwise comparison. Significance of the results obtained was judged at the 5% level [39].

### 4.6. Statistical Analysis

Data from biochemical analysis and LM histomorphometric analyses were entered into IBM SPSS Statistics version 20.0 and analyzed (Armonk, NY, USA: IBM Corp.). Continuous data were tested for normality by the Shapiro–Wilk test. Quantitative data were expressed as range (minimum and maximum), mean, and standard deviation. One-way ANOVA test was used for comparing the different studied groups, followed by a post hoc test (Tukey) for pairwise comparisons. The significance of the results obtained was judged at the 5% level [39].

## 5. Conclusions

The histological, biocompatibility, and biochemical assessments demonstrated that while luteolin (LU) and chitosan (CH) exhibited osteoconductive and regenerative properties, their standalone application was less practical at promoting bone formation than the innovative LU-loaded chitosan vesicular system (LU-CHV). Remarkably, LU-CHV nearly restored bone healing parameters to levels approaching those of the control group, indicating a substantial enhancement in regenerative capacity. This near-complete normalization of histomorphometric and biochemical markers underscores the synergistic effect of controlled drug delivery and localized modulation of inflammatory and oxidative pathways. Such findings highlight the therapeutic potential of nanocarrier-based strategies in oral surgery, particularly for conditions associated with impaired bone regeneration, such as diabetes and obesity. By improving drug bioavailability and maintaining local bioactivity, LU-CHV represents a promising advancement in regenerative oral therapies and socket-healing management.

## 6. Study Limitations

While the study results demonstrate the significant regenerative potential of the LU-CHV, several limitations must be acknowledged:

1. The study utilized a rat model, which has a faster metabolic rate and different mechanical loading patterns compared to the human alveolar bone.
2. The RANKL/OPG modulation is proposed as the primary mechanism, but direct molecular quantification, as RT-qPCR for gene expression, was not performed to confirm the exact signaling pathways.
3. The six-week observation period captures the critical healing phases. However, the long-term stability of the regenerated bone remains to be investigated.

## Figures and Tables

**Table 1:** Body Weight Changes During Obesity Induction.

Group	Baseline Body Weight (g)	Pre-STZ Body Weight	% Weight Gain
Control	185 ± 8	240 ± 10	29.7%
HFD + STZ	186 ± 7	298 ± 11 *	60.2%

* Significantly different from the Control group (*p* < 0.05).

**Table 2:** Fasting Blood Glucose Levels (mg/dL).

Group	Baseline BGL	Post-STZ	End of Study BGL
Control	92 ± 6	95 ± 7	98 ± 8
HFD + STZ	101 ± 7	312 ± 28 *	298 ± 35 *

* Significantly different from the Control group (*p* < 0.05). Diabetes was confirmed when BGL > 250 mg/dL.

**Table 3:** Colloidal features of the prepared LU vesicles.

Formulation	% EE	Particle Size (nm)	PDI	Zeta Potential (mV)
LU-blank	60 ± 2.18	88.92 ± 1.14	0.22 ± 0.05	−20.05 ± 1.56
LU-CHV	92.33 ± 1.14	220.71 ± 2.70	0.31 ± 0.13	23.60 ± 2.78
LU-CHV *	91.78 ± 0.14	223.51 ± 3.26	0.32 ± 0.08	24 ± 2.45

* LU-CHV after storage for 6 months at 4 °C.

**Table 4:** Comparison between all groups according to light microscopic results of bone profile at the end of the experiment.

Feature	Group I	Group II	Groups III and IV	Group V
**Primary Filling**	Mature NB/BM	Fat Cells/Thin B	Mixed GT/Thin NB	Dense NB/Vascular BM
**Osteocyte Profile**	Normal	Minimal	Large/Prominent	High Density/Large OS
**Healing Quality**	Optimal (Baseline)	Delayed/Fibrotic	Intermediate	Accelerated

**Table 5:** RANKL//OPG Ratio.

**(A) After two weeks**			
**Group**	**RANKL/(pg/mg)**	**OPG (pg/mg)**	**RANKL//OPG Ratio**
**Group I (negative control)**	175.3	616.0	**0.28**
**Group II (positive control)**	393.8	527.1	**0.75**
**Group III (LU-treated)**	301.9	589.4	**0.51**
**Group IV (CHV-treated)**	310.8	570.6	**0.54**
**Group V (LU-CHV-treated)**	215.8	663.6	**0.33**
**(B) After six weeks**			
**Group**	**RANKL/(pg/mg)**	**OPG (pg/mg)**	**RANKL//OPG Ratio**
**Group I (negative control)**	144.6	700.6	**0.21**
**Group II (positive control)**	264.3	560.5	**0.47**
**Group III (LU-treated)**	174.3	644.4	**0.27**
**Group IV (CHV-treated)**	186.6	639.9	**0.29**
**Group V (LU-CHV-treated)**	149.9	712.3	**0.21**

## Data Availability

The original contributions presented in the study are included in the article and supplementary material, further inquiries can be directed to the corresponding author/s.

## Supplementary Materials

The following supporting information can be downloaded at: https://www.mdpi.com/article/10.3390/ph19030506/s1, Table S1. Comparison between all experimental groups according to biocompatibility test results. Table S2. Comparison between all experimental groups according to Inflammatory Cytokines (IL-6, TNF-α, and IL-1β). Table S3. Comparison among all experimental groups for Osteocalcin, OPG, and RANKL biomarkers. Table S4. Comparison between all experimental groups based on histomorphometric analysis of bone surface area (%). Table S5. Comparison among all experimental groups based on histomorphometric analysis of Marrow Space Percentage. Table S6. Comparison between all experimental groups according to histomorphometric analysis of Inflammatory Cell Count (%).

## Abbreviations

The following abbreviations are used in this manuscript:

LU	Luteolin
CH	Chitosan
CHV	Chitosan vesicles
EE	Entrapment efficiency
PS	Particle size
ZP	Zeta potential
TNF-α	Tumor necrosis factor-alpha
TGF-β1	Transforming growth factor-beta 1
IL-6	Interleukin-6
DM	Diabetes mellitus
OPG	Osteoprotegerin
PDI	Polydispersity index
TEM	Transmission electron microscope
