# Supplementary material for: Tailored Phytochitosomes as Targeted Nanotherapy for Alveolar Bone Regeneration in Diabetic Obese Rats
Source: Pharmaceuticals (Basel). 2026 Mar 19;19(3):506. doi: 10.3390/ph19030506 (PMC13029201; doi:10.3390/ph19030506)
Supplement: Supplementary file 1 [file pharmaceuticals-19-00506-s001.zip › pharmaceuticals-4186008-supplementary.pdf]

Table S1. Comparison between all experimental groups according to biocompatibility test

	Epidermal Thickness (μm)	Collagen Area Fraction (%)	Subcutaneous Depth (μm)
Group I	94.5±5.2	43.4±2.1	193.4±22.8
Group II	51.2±4.8	18.7±3.5	290.1±20.4
Group III	65.8±6.1	24.2±4.2	245.8±18.2
Group IV	82.4±7.5*	32.8±5.1*	220.5±12.1*
Group V	95.7± 8.1**	41.5 ±5.5**	185.3±15.4**

results

(*) and (**) statistical significance at $p < 0.05$ and $p < 0.001$ respectively, compared to Group II.
P1 (Group II vs. Group I) *** ($p < 0.001$)
P2 (Group III vs. Group II) * ($p < 0.05$)
P3 (**Group IV vs. Group II) * (**$p < 0.05$)
P4 (**Group V vs. Group II)** ***($p < 0.001$)
P5 (Group V vs. Group I) ns

Table S2. Comparison between all experimental groups according to Inflammatory Cytokines (IL-6, TNF-α, and IL-1β).

		Group I (negative control)	Group II (positive control)	Group III (LUT-treated)	Group IV (HAS-treated)	Group V (LUT-HAS2-treated)	F	p
**IL-6 (pg/mg protein)**	**After 2 weeks**							
	Min. – Max.	22.0 – 41.0	65.0 – 99.0	52.0 – 84.0	59.0 – 80.0	35.0 – 55.0	45.468*	<0.001*
	Mean ± SD.	32.50 ± 5.93	83.63[a] ± 10.90	65.50[ab] ± 10.53	68.25[ab] ± 7.36	43.13[bcd] ± 7.22		
	**p_0**		<0.001*	<0.001*	<0.001*	0.122		
	**p_1**			0.002*	0.009*	<0.001*		
	**Sig. bet. Groups.**	**p_2=0.968, p_3<0.001*, p_4<0.001***						
	**After 6weeks**							
	Min. – Max.	18.0 – 28.0	41.0 – 64.0	29.0 – 49.0	37.0 – 52.0	21.0 – 38.0	29.421*	<0.001*
	Mean ± SD.	22.50 ± 3.66	52.13[a] ± 6.96	39.63[ab] ± 7.78	42.88[ab] ± 5.38	28.25[bcd] ± 6.16		
	**p_0**		<0.001*	<0.001*	<0.001*	0.352		
	**p_1**			0.002*	0.037*	<0.001*		
	**Sig. bet. Groups.**	**p_2=0.827, p_3=0.006*, p_4<0.001***						
	**p_5**	0.001*	<0.001*	<0.001*	<0.001*	0.001*		
**TNF-alpha (pg/mg protein)**	**After 2 weeks**							
	Min. – Max.	20.0 – 40.0	75.0 – 105.0	50.0 – 75.0	51.0 – 79.0	39.0 – 59.0	54.792*	<0.001*
	Mean ± SD.	31.38 ± 6.09	89.88[a] ± 10.09	63.38[ab] ± 8.88	65.38[ab] ± 8.90	47.38[abcd] ± 7.19		
	**p_0**		<0.001*	<0.001*	<0.001*	0.004*		
	**p_1**			<0.001*	<0.001*	<0.001*		
	**Sig. bet. Groups.**	**p_2=0.989, p_3=0.004*, p_4=0.001***						
	**After 6 weeks**							
	Min. – Max.	18.0 – 31.0	42.0 – 63.0	29.0 – 45.0	28.0 – 47.0	19.0 – 36.	24.405*	<0.001*
	Mean ± SD.	24.25 ± 4.62	52.13[a] ± 7.75	36.38[ab] ± 5.40	37.13[ab] ± 7.06	26.50[bcd] ± 6.26		
	**p_0**		<0.001*	0.004*	0.002*	0.952		
	**p_1**			<0.001*	<0.001*	<0.001*		
	**Sig. bet. Groups.**	**p_2=0.999,p_3=0.027*,p_4=0.015***						
	**p_5**	0.020*	<0.001*	<0.001*	<0.001*	<0.001*		
**IL-1B (pg/mg protein)**	**After 2 weeks**							
	Min. – Max.	19.0 – 37.0	49.0 – 75.0	38.0 – 58.0	38.0 – 66.0	26.0 – 41.0	26.163*	<0.001*
	Mean ± SD.	26.50 ± 6.12	61.88[a] ± 10.08	46.63[ab] ± 7.44	48.38[ab] ± 8.47	32.50[bcd] ± 5.68		
	**p_0**		<0.001*	<0.001*	<0.001*	0.536		
	**p_1**			0.003*	0.011*	<0.001*		

**Sig. bet. Groups.**	**p_2=0.991, p_3=0.007*, p_4=0.002***						
**After 6 weeks**							
Min. – Max.	12.0 – 21.0	29.0 – 55.0	20.0 – 38.0	25.0 – 38.0	18.0 – 31.0	17.831*	<0.001*
Mean ± SD.	17.13 ± 3.0	39.0^{a} ± 8.11	29.88^{ab} ± 5.96	30.88^{ab} ± 4.88	23.25^{b} ± 4.40		
**p_0**		<0.001*	<0.001*	<0.001*	0.199		
**p_1**			0.018*	0.044*	<0.001*		
**Sig. bet. Groups.**	**p_2=0.996, p_3=0.141, p_4=0.066**						
**p_5**	0.002*	<0.001*	<0.001*	<0.001*	0.003*		

SD: Standard deviation, F: F for the One-way ANOVA test, pairwise comparison between. Every two groups were done using the Post Hoc Test (Tukey), p: p-value for comparing the studied groups, p0: p-value for comparing the negative control and each of the other groups, p1: p-value for comparing the positive control and each other group, p2: p-value for comparing LU-treated and CHV-treated, p3: p-value for comparing LU-treated and LU-CHV-treated, p4: p-value for comparing CHV-treated and LU-CHV-treated, p5: p-value for comparing after two weeks and after six weeks in each group, *: Statistically significant at $p \le 0.05$, a: Significant with negative control, b: Significant with positive control, c: Significant with LU-treated, d: Significant with CHV-treated.

Table S3. Comparison among all experimental groups for Osteocalcin, OPG, and RANKL biomarkers.

		Group I (negative control)	Group II (positive control)	Group III (LU-treated)	Group IV (CHV-treated)	Group V (LU-CHV-treated)	F	p
**Osteocalcin (ng/mg protein)**	***After two weeks***							
	Min. – Max.	35.0 – 61.0	21.0 – 36.0	25.0 – 44.0	22.0 – 39.0	37.0 – 62.0	10.187^{*}	$<0.001^{*}$
	Mean ± SD.	47.38 ± 8.60	29.13^{a} ± 5.59	35.63^{a} ± 6.37	31.25^{a} ± 5.95	45.25^{bd} ± 9.16		
	**p_0**		$<0.001^{*}$	0.021^{*}	0.001^{*}	0.977		
	**p_1**			0.398	0.977	0.001^{*}		
	**Sig. bet. Groups.**			**p_2=0.750, p_3=0.084, p_4=0.004^{*}**				
	After six weeks							
	Min. – Max.	52.0 – 82.0	29.0 – 44.0	31.0 – 59.0	33.0 – 59.0	60.0 – 82.0	26.110^{*}	$<0.001^{*}$
	Mean ± SD.	69.63 ± 10.06	37.0^{a} ± 5.55	43.38^{a} ± 9.84	46.13^{a} ± 10.51	73.38^{bcd} ± 8.70		
	**p_0**		$<0.001^{*}$	$<0.001^{*}$	$<0.001^{*}$	0.922		
	**p_1**			0.632	0.286	$<0.001^{*}$		
	**Sig. bet. Groups.**			**p_2=0.974, p_3<0.001^{*}, p_4<0.001^{*}**				
	**p_5**	$<0.001^{*}$	0.013^{*}	0.083	0.005^{*}	$<0.001^{*}$		
**OPG (pg/mg protein)**	***After two weeks***							
	Min. – Max.	510.0 – 680.0	460.0 – 630.0	500.0 – 650.0	480.0 – 710.0	570.0 – 750.0	4.890^{*}	0.003^{*}
	Mean ± SD.	616.0 ± 59.17	527.1 ± 58.63	589.4 ± 51.30	570.6 ± 83.60	663.6^{b} ± 68.18		
	**p_0**		0.070	0.923	0.636	0.593		
	**p_1**			0.330	0.671	0.002^{*}		
	**Sig. bet. Groups.**			**p_2=0.978, p_3=0.175, p_4=0.052**				
	After six weeks							
	Min. – Max.	620.0 – 810.0	470.0 – 670.0	590.0 – 730.0	555.0 – 720.0	610.0 – 754.0	9.515^{*}	$<0.001^{*}$
	Mean ± SD.	700.6 ± 66.25	560.5^{a} ± 66.30	644.4^{b} ± 45.63	639.9 ± 48.33	712.3^{b} ± 45.94		
	**p_0**		$<0.001^{*}$	0.272	0.205	0.993		
	**p_1**			0.035^{*}	0.051	$<0.001^{*}$		
	**Sig. bet. Groups.**			**p_2=1.000, p_3=0.125, p_4=0.089**				
	**p_5**	0.017^{*}	0.304	0.040^{*}	0.062	0.117		
**RA**	***After two weeks***							

Min. – Max.	150.0 – 210.0	350.0 – 445.0	260.0 – 370.0	280.0 – 360.0	160.0 – 270.0	57.226*	<0.001*
Mean ± SD.	175.3 ± 19.65	393.8^{a} ± 31.02	301.9^{ab} ± 38.17	310.8^{ab} ± 32.23	215.8^{bcd} ± 36.12		
**p_0**		<0.001*	<0.001*	<0.001*	0.108		
**p_1**			<0.001*	<0.001*	<0.001*		
**Sig. bet. Groups.**	**p_2=0.981, p_3<0.001*, p_4<0.001***						
After six weeks							
Min. – Max.	137.0 – 159.0	240.0 – 290.0	155.0 – 192.0	165.0 – 210.0	129.0 – 177.0	91.813*	<0.001*
Mean ± SD.	144.6 ± 7.25	264.3^{a} ± 18.25	174.3^{ab} ± 12.83	186.6^{ab} ± 13.91	149.9^{bcd} ± 16.27		
**p_0**		<0.001*	0.002*	<0.001*	0.946		
**p_1**			<0.001*	<0.001*	<0.001*		
**Sig. bet. Groups.**	**p_2=0.422,p_3=0.013*,p_4<0.001***						
**p_5**	0.003*	<0.001*	<0.001*	<0.001*	<0.001*		

SD: Standard deviation, F: F for the One-way ANOVA test, pairwise comparison between. Every two groups were done using the Post Hoc Test (Tukey), p: p-value for comparing the studied groups, p0: p-value for comparing the negative control and each of the other groups, p1: p-value for comparing the positive control and each different group, p2: p-value for comparing between LU-treated and CHV-treated, p3: p-value for comparing between LU-treated and LU-CHS, p4: p-value for comparing between CHV-treated and LU-CHV-treated, p5: p-value for comparing between After two weeks and after six weeks in each group, *: Statistically significant at p ≤ 0.05, a: Significant with negative control, b: Significant with positive control, c: Significant with LU-treated, d: Significant with CHV-treated.

Table S4. Comparison between all experimental groups based on histomorphometric analysis of bone surface area (%).

Group	Two Weeks (Mean ± SD)	P_0	P_1	Six Weeks (Mean ± SD)	P_0	P_1	p
**Group I (negative control)**	48.6 ± 3.2	<0.0001*		72.4 ± 4.1	<0.0001*		a
**Group II (positive control)**	14.8 ± 2.1	<0.0001*		26.5 ± 3.0	<0.0001*		b
**Group III (LU-treated)**	25.7 ± 2.9	<0.0001*	<0.0001*	41.3 ± 3.6	<0.0001*	<0.0001*	c
**Group IV (CHV-treated)**	33.9 ± 3.1	0.0002	<0.0001*	55.7 ± 4.0	0.2069	<0.0001*	d
**Group V (LU-CHV-treated)**	45.5 ± 3.4	<0.0001*	<0.0001*	69.1 ± 3.8	0.2069	<0.0001*	a*
**Sig. bet. Groups**	**P2=<0.001*, p3<0.001*, p4<0.001***			**P2=<0.001*, p3<0.001*, p4<0.001***			

p: p-value for comparing the studied groups, p0: p-value for comparing the negative control and each of the other groups, p1: p-value for comparing the positive control and each of the different groups, p2: p-value for comparing LU-treated and CHV-treated, p3: p-value for comparing LU-treated and LU-CHV, p4: p-value for comparing CHV-treated and LU-CHV-treated, p5: p-value for comparing after two weeks and after six weeks in each group, a = not significantly different from Group V, b = considerably lower than all groups, c = markedly higher than Group II, lower than IV & V, d = significantly higher than Groups II & III, lower than I & V, a* = no significant difference between Group V and Group I (p > 0.05).

Table S5. Comparison among all experimental groups based on histomorphometric analysis of Marrow Space Percentage.

Group	Two Weeks (Mean ± SD)	P_0	P_1	Six Weeks (Mean ± SD)	P_0	P_1	p
Group I (negative control)	22.4 ± 2.1	<0.0001*		10.8 ± 1.8	<0.0001*		a
Group II (positive control)	58.7 ± 3.9	<0.0001*		41.5 ± 3.2	<0.0001*		d
Group III (LU-treated)	45.2 ± 3.1	<0.0001*	<0.0001*	29.4 ± 2.8	<0.0001*	<0.0001*	c
Group IV (CHV-treated)	33.1 ± 2.7	0.0002*	<0.0001*	18.9 ± 2.2	0.0002*	<0.0001*	b
Group V (LU-CHV-treated)	25.7 ± 2.3	0.8000	<0.0001*	12.6 ± 1.9	0.6789	<0.0001*	a
Sig. bet. Groups	P2=<0.001*, p3<0.001*, p4<0.001*			P2= 0.0012, p3<0.001*, p4<0.001*			

p: p-value for comparing the studied groups, p0: p-value for comparing the negative control and each of the other groups, p1: p-value for comparing the positive control and each of the different groups, p2: p-value for comparing LU-treated and CHV-treated, p3: p-value for comparing LU-treated and LU-CHV, p4: p-value for comparing CHV-treated and LUT-CHV-treated, p5: p-value for comparing after two weeks and after six weeks in each group, a: Best (no significant difference between G1 and G5), d: Worst (significantly higher spaces than all groups), b/c: Intermediate levels with gradual improvement.

Table S6. Comparison between all experimental groups according to histomorphometric analysis of Inflammatory Cell Count (%):

Group	Two Weeks (Mean ± SD)	P_0	P_1	Six Weeks (Mean ± SD)	P_0	P_1	p
**Group I (negative control)**	9.6 ± 1.5	<0.0001*		4.2 ± 0.9	<0.0001*		a
**Group II (positive control)**	39.4 ± 3.8	<0.0001*		26.1 ± 2.7	<0.0001*		d
**Group III (LU-treated)**	28.7 ± 2.9	<0.0001*	<0.0001*	16.4 ± 1.9	<0.0001*	<0.0001*	c
**Group IV (CHV-treated)**	20.3 ± 2.1	<0.0001*	<0.0001*	9.8 ± 1.4	0.0055	<0.0001*	b
**Group V (LU-CHV-treated)**	11.5 ± 1.7	0.6420	<0.0001*	5.3 ± 1.0	0.9973	<0.0001*	a
**Sig. bet. Groups**	**P2=0.0101, p3<0.001*, p4<0.001***			**P2<0.001*, p3<0.001*, p4<0.0133***			

p: p-value for comparing the studied groups, p_0: p-value for comparing the **negative control** and each of the other groups, p_1: p-value for comparing the **positive control** and each of the different groups, p_2: p-value for comparing **LU-treated** and **CHV-treated**, p_3: p-value for comparing **LU-treated** and **LU-CHV**, p_4: p-value for comparing **CHV-treated** and **LU-CHV-treated**, p_5: p-value for comparing **after two weeks** and **after six weeks** in each group, Group II shows the highest inflammation., Groups III and IV show progressive improvement., Group V almost matches healthy controls (Group I)
